# Evaluation of the cargo contents and potential role of extracellular vesicles in osteoporosis

**DOI:** 10.18632/aging.203264

**Published:** 2021-08-10

**Authors:** Jifeng Xu, Yu Chen, Dongsheng Yu, Li Zhang, Xiaofan Dou, Gang Wu, Yaping Wang, Shuijun Zhang

**Affiliations:** 1 Department of Orthopaedic Surgery, Zhejiang Provincial People’s Hospital, People’s Hospital of Hangzhou Medical College, Hangzhou, Zhejiang 310014, PR China; 2 Department of Orthopaedic Surgery, the Second People’s Hospital of Fuyang, Hangzhou, Zhejiang 311404, PR China; 3 Department of Cardiology, 2nd Affiliated Hospital, School of Medicine, Zhejiang University, Zhejiang 310009, PR China

**Keywords:** osteoporosis, extracellular vesicles, microRNAs, Wnt

## Abstract

Osteoporosis is a common aging-related disease diagnosed primarily using bone mineral density (BMD). Extracellular vesicles (EVs) remain unexplored in the context of osteoporosis. Towards this, EVs were isolated from plasma of a discovery cohort with 8 non-osteoporotic and 8 osteoporotic individuals, and nanoparticle tracking analysis (NTA) revealed a significantly higher EV concentration in osteoporotic individuals (*P* = 0.003). Moreover, EVs concentration showed a linear correlation with bone mineral density (BMD) values (linear correlation coefficient *r* = 0.9542, deviation from zero, *p* < 0.001). Results using a mouse model of osteoporosis confirmed that the number of EVs in mice from hindlimb unloading group was significantly higher than that from the age-matched control group (*p* = 0.015). TaqMan Real-Time PCR demonstrated that miR-335-5p, -320a, -483-5p, and miR-21-5p, were significantly higher expressed in osteoporotic patients compared with non-osteoporotic individuals. Quantitative real-time PCR shown that Wnt1, Wnt5a, Wnt7a, and Wnt9a mRNAs were lower expressed in osteoporosis derived EVs. *In vitro* functional assay indicated that osteoporosis derived EVs resulted in reduced mineralization in SaOS-2 cells. In conclusion, these results suggest that osteoporosis increased the secretion of EVs which carry higher expression of miRNAs and decreased expression of Wnt signals, further decreased the mineralization capacity in human osteoblasts.

## INTRODUCTION

Osteoporosis is an ageing-related disease, clinically featured by bone loss and increased risk of fracture [1]. The prevalence of osteoporosis usually ranges from 2 to 8% among males and 9 to 38% among females [2]. In China, the incidence of osteoporosis has increased over the past several years, affecting nearly 1/3 of people over 50 years old [3]. The majority of osteoporosis cases occur due to an imbalance between bone resorption and bone formation [4]. There are well documented studies that suggest that microRNAs (miRNAs), non-coding RNAs, are critical regulators of osteoblast-mediated bone formation and remodelling [5]. Deregulation of miRNA expression is clearly associated with bone resorption, bone formation and osteoporosis [5–8]. The selective expression of miR-34c in osteoblasts from ageing mice resulted in defects in osteoblast proliferation and mineralization [9]. It was reported that lupus patients with higher level of miR-148a expression shown reduced bone mineral density (BMD), which was consistent with the finding that increased expression level of miR-148a lead to pro-osteoclastic effects [10]. Additionally, miR-133a expression is linked to lower BMD [11].

Extracellular vesicles (EVs) are characterized as membrane-bound nanoparticles (30–400 nm) [12]. Specifically, circulating EVs are differentially changing in size [12]. In healthy bone, exosomes can transmit molecules involved in both bone synthesis and resorption and are also involved in bone remodelling and even extracellular matrix mineralization. Till now, it remains elusive of EVs contents and function in osteoporosis. In this study, we examined the concentration, cargo and function of extracellular vesicles in osteoporosis. We evaluated the effects of osteoporosis on the EV profile and on alterations in miRNAs and the Wnt signalling pathway in osteoporosis-derived EVs. We further examined the potential roles of osteoporosis-related EVs in regulating mineralization in osteoclast cells.

## RESULTS

### Increased EV concentrations in osteoporosis

**Figure 1:**
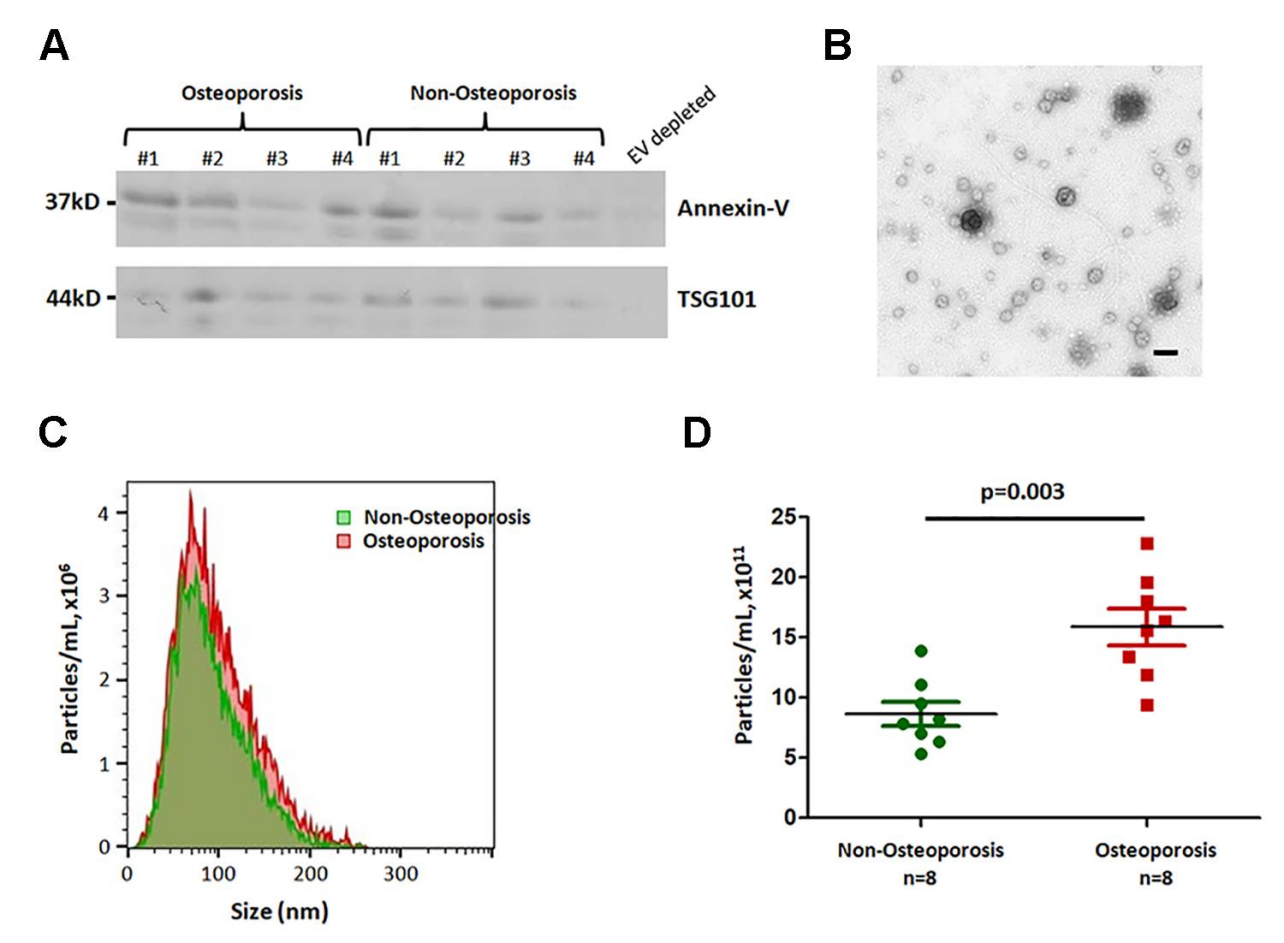
**Characterization and concentration analysis of extracellular vesicles.** (**A**) Plasma-derived EVs isolated from four individuals with osteoporosis, four individuals without osteoporosis, one EV-depleted plasma sample were subjected to SDS-PAGE and probed for EV-enriched proteins, Annexin-V and TSG101. (**B**) Electron microscopy of EVs. Scale bar = 500 nm. (**C**) and (**D**) Nanoparticle tracking analysis (NTA). The area under the curve in (**C**) is shown in (**D**).

To examine the role of EVs in osteoporosis, plasma EVs were isolated by subjecting to sequential low- and high-speed centrifugation. Exosome markers Annexin-V and TSG101 were found in all EV samples (Figure 1A). In addition, we observed the intact round vesicles of 50–200 nm under the electron microscopy (Figure 1B). Nanoparticle tracking analysis confirmed that the EVs were 50–150 nm (a peak around 83 nm) spherical particles with a complete membrane structure (Figure 1C), demonstrating that these vesicles are characteristic of exosomes.

The concentration and size of EVs were compared between 8 non-osteoporotic and 8 osteoporotic individuals. Table 1 showed the patient demographic data. Results shown that there was higher concentration of EV in osteoporotic patients compared to non-osteoporotic controls (*P* = 0.003; Figure 1D).

**Table 1:** Summary of characteristics of clinical samples enrolled in the pilot study.

**Clinical Category**	**Non-Osteoporosis Control**	**Osteoporosis**
Female/Male	4/4	4/4
Mean age years (Mean ± SD)	65 ± 7.8	66.7 ± 9.5
BMD (g/cm^2^) Mean ± SD	0.95 ± 0.03	0.68 ± 0.08
T-Score Lumbar Spine (L2-L4) Mean ± SD	0.56 ± 1.33	−2.53 ± 0.88

A differential ultracentrifugation protocol [13] was employed to further evaluate the EV concentration. Higher concentration of EVs was found in osteoporotic subjects when EVs were spin down at the speed of 10,000 × g and 120,000 × g (Supplementary Figure 1).

### Plasma EVs concentrations correlate to BMD and are gender independent in osteoporotic patients

**Figure 2:**
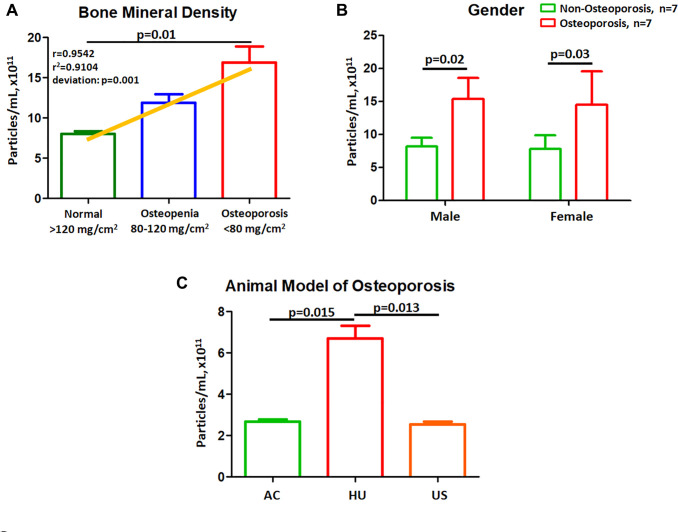
**Plasma EVs concentrations correlate to BMD and gender.** (**A**) EV concentration grouped by obtained BMD value. Linear regression analysis was performed for EV concentration using a cohort of 7 normal controls, 7 Osteopenia and 7 Osteoporotic patients. (**B**) EV concentration in male and female were compared between osteoporotic and non-osteoporotic individuals. (**C**) Effects of hindlimb unloading and salubrinal on EV concentration in a mouse model of osteoporosis.

We further test the correlation of EVs with BMD by using a cohort of 7 normal controls, 7 Osteopenia and 7 Osteoporotic patients. Results shown EVs concentration had a linear correlation with BMD values (*r* = 0.9542, *p* < 0.001) (Figure 2A). We further recruited an independent cohort to evaluate the effect of gender on EV concentration (Table 2). There were no significant differences in EVs concentrations (Male, *p* = 0.02; Female, *p* = 0.03, Figure 2B) comparing to non-osteoporotic and osteoporotic males. We further confirm the results using a mouse model of osteoporosis [14], mice were divided into 3 groups: AC, HU, and US. Results shown that the number of extracellular vesicles in HU mice was higher than AC (both *p* = 0.015, Figure 2C). US mice had reduced numbers of EVs compared to that of HU mice (both *p* = 0.013, Figure 2C).

**Table 2:** Demographical characteristics of osteoporotic patients enrolled for gender effect analysis on EV profiles.

	**Female osteoporotic**	**Female non-osteoporotic**
Included patients (N)	7	7
Age (y), mean (range)	76.5 (68–85)	72.5 (65–84)
Body mass index (kg/m^2^) ± SD	23.8 ± 2.1	24.2 ± 2.5
main diagnosis (beside osteoporosis)	femoral neck fracture	–
Volumetric BMD by qCT (mg/cm^3^) ± SD	66.1 ± 14.5	225.4 ± 35.8
	**Male osteoporotic**	**Male non-osteoporotic**
Included patients (N)	7	7
Age (y), mean (range)	75.2 (68–82)	69.9 (65–78)
Body mass index (kg/m^2^) ± SD	24.6 ± 3.1	26.1 ± 4.3
main diagnosis (beside osteoporosis)	femoral neck fracture	–
Volumetric BMD by qCT (mg/cm^3^) ± SD	36.4 ± 36.7	233.1 ± 45.1

No statistical differences concerning demographics in between the groups exist. Only the “volumetric BMD by qCT” values are significantly different between osteoporotic and non-osteoporotic patients (*p* < 0.05).

### MicroRNAs expression profile was dysregulated in osteoporosis derived EVs

**Figure 3:**
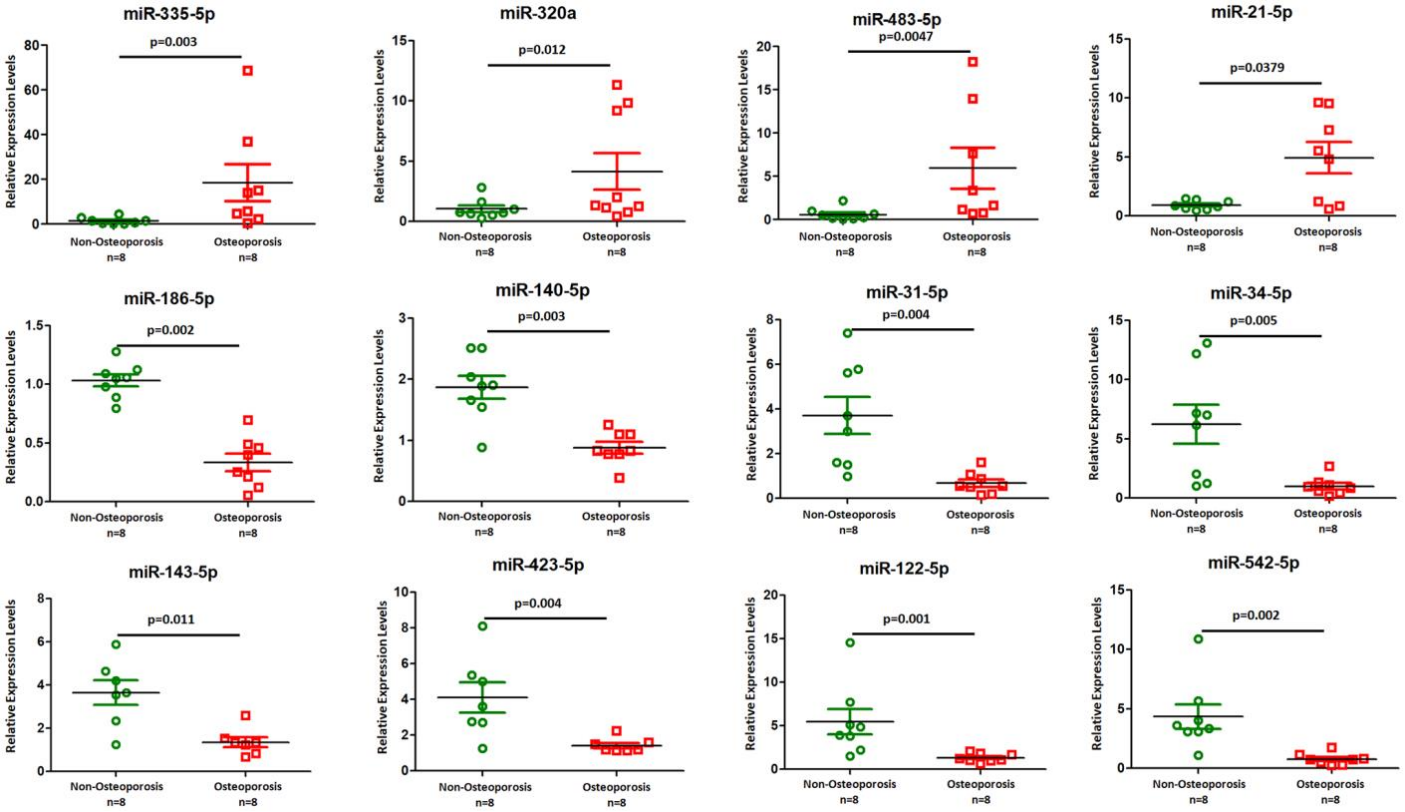
**TaqMan real-time PCR to examine the expression levels of miR-335-5p, -320a, -483-5p, -21-5p, -186-5p, -140-5p, -31-5p, -34-5p, -143-5p, -423-5p, -122-5p, and miR-542-5p in plasma derived EVs from eight osteoporotic patients compared with eight non-osteoporotic individuals.** Data shown are as mean ± SEM.

We chose TaqMan PCR to study the microRNA expression and potential roles of the small non-coding RNAs especially miRNAs in EVs. Twelve miRNAs, including miR-335-5p, -320a, -483-5p, -21-5p, -186-5p, -140-5p, -31-5p, -34-5p, -143-5p, -423-5p, -122-5p, and miR-542-5p, were selected according to their significance in previous observations and biological significance [5, 7, 8]. We found that four miRNAs, including miR-335-5p, -320a, -483-5p, and miR-21-5p, were significantly higher expressed in osteoporotic patients compared with non-osteoporotic individuals (Figure 3). Furthermore, eight miRNAs including miR-186-5p, -140-5p, -31-5p, -34-5p, -143-5p, -423-5p, -122-5p, and miR-542-5p represented a significantly lower abundance in osteoporotic patients compared with non-osteoporotic individuals (Figure 3). These findings indicated that dysregulated vesicle non-coding small RNAs may involve in osteoporosis development.

### Wnt containing EVs in osteoporosis

**Figure 4:**
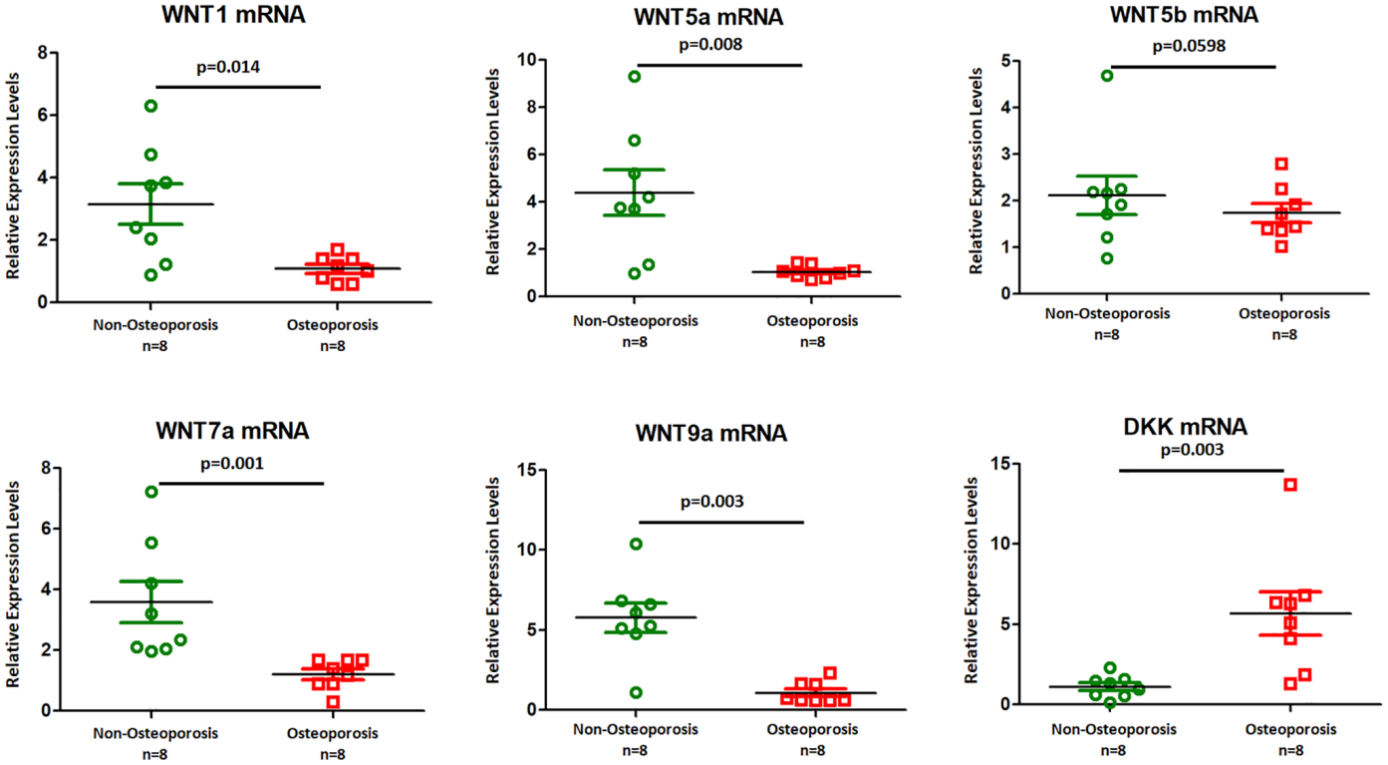
**Wnt ligands mRNAs were higher in EVs isolated from osteoporotic patients.** Six mRNAs (Wnt1, Wnt5a, Wnt5b, Wnt7a, Wnt9a, and DKK) were selected for qRT-PCR experiments using eight osteoporotic patients compared with eight non- osteoporotic individuals. Data represent mean ± SEM from three independent experiments.

Canonical Wnt signaling was previously implicated in osteoporosis [15]. We further sought to examine the role of EVs in osteoporosis. Quantitative real-time PCR shown that Wnt1, Wnt5a, Wnt7a, and Wnt9a mRNAs were lower expressed in osteoporosis derived EVs (Figure 4), while DKK mRNA was up regulated in osteoporosis compared to non-osteoporotic individuals. There was no significant change in Wnt5b mRNA level (Figure 4). Our results suggest that Wnt signal as a potential regulator of osteoporosis development.

### Treatment of osteoporosis EVs reduced the mineralization

**Figure 5:**
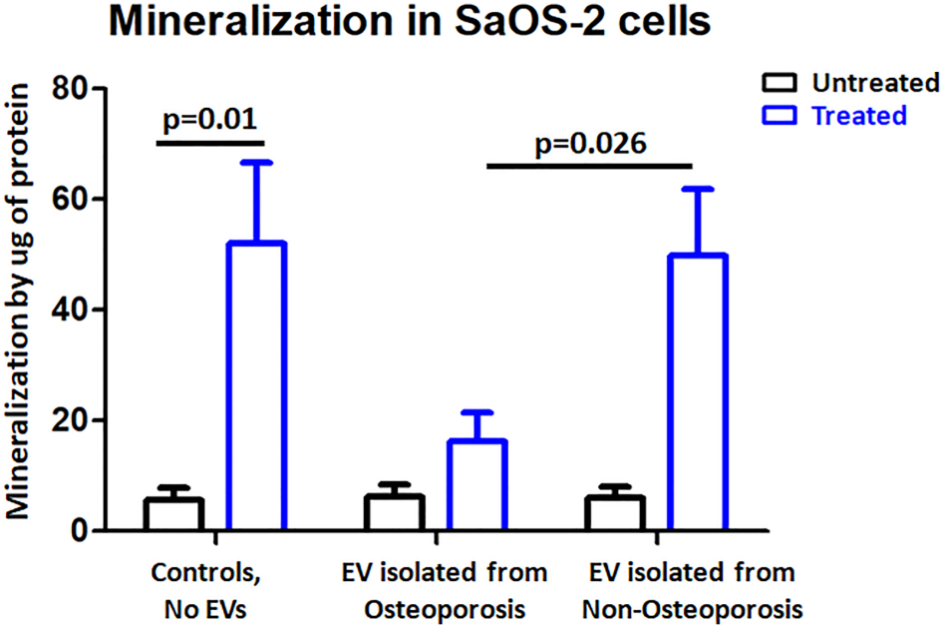
**Osteoporosis derived EVs resulted in reduced mineralization in SaOS-2 cells.** Mineralization quantification in control cells and EV-treated cells in either the presence of osteogenic factors (treated) or the absence (untreated). Bar-plot of *n* = 3 independent experiments ± SEM to quantify mineralization; Results were analyzed by one-way analysis of variance (ANOVA) and Bonferroni post hoc tests.

To investigate the role of EVs in osteoporosis, we tested the mineralization in SaOS-2 cells after treatment with osteoporosis derived EVs. The positive control shown that control cells had a ten-fold increase in mineralization. However, induced mineralization was severely impaired after adding osteoporosis derived EVs (Figure 5), non-osteoporosis derived EVs did not reduce the mineralization. These findings suggest that osteoporosis derived EVs influence mineralization capacity in human osteoblasts.

## DISCUSSION

Osteoporosis is the most common systemic bone disease characterized by a rapid decline in bone mass [16, 17]. In this study, we investigated the effects of osteoporosis on EV profiles and alterations in osteoporosis-related miRNAs and the Wnt signalling pathway. The results of our study revealed a remarkably higher concentration of EV in osteoporotic subjects than in non-osteoporotic individuals (*P* = 0.003). In addition, plasma EV concentrations were found to be related to BMD, and this effect was gender independent in osteoporotic patients. More importantly, significantly more extracellular vesicles were present in a mouse model of osteoporosis than in control mice. EVs are heterogeneous nanosized vesicles, and their cargo contains nucleic acids and proteins on the surface or in the lumen. EV structure allows for the transfer of EV cargo to specific acceptor cells; thus, through this mechanism, EVs communicate between cells during osteoporosis. Patients with type 1 diabetes tend to have the pro-osteogenic properties of stem cells-derived exosomes, consistent with the pathogenesis of diabetes-related osteoporosis [18]. In addition, the systemic injection of stem cell derived EVs can inhibit osteoporosis by promoting osteoblastic bone formation [19].

miRNAs are involved in bone formation and resorption [5, 7, 8]. Claudine Seeliger et al. identified that serum miRNAs and tissue miRNAs are associated with osteoporotic fractures [20]. Hongqiu Li et al. detected the plasma expression levels of miR-21, miR-133a and miR-146a that related with BMD in osteoporosis [21]. miR-140-3p and miR-214 were reduced in the animal models of osteoporotic fractures, while miR-148a and miR-218 were associated with osteoclastogenesis [22]. miR-21 was found to be up-regulated in osteoporotic individuals and shown antiosteogenic effect by inhibiting SMAD7 protein and subsequently down regulating ALP, OCN, and RUNX2 [23]. miR-155 found in endothelial progenitor cell-derived exosomes could inhibits osteoporosis by blocking osteoclast induction [24]. In addition, miR-125 could promote bone regeneration by inducing angiogenesis during distraction osteogenesis [25]. More importantly, several miRNAs were validated to regulate Wnt signaling. For example, miR-152-3p and miR-335 could target Dickkopf-1 [26, 27], while miR-22-3p and miR-34a-5p specifically target WNT1 [28, 29]. The results of our study demonstrated that the miRNA expression profile was dysregulated in osteoporosis-derived EVs. We found that four miRNAs, including miR-335-5p, miR-320a, miR-483-5p, and miR-21-5p, were significantly more highly expressed in osteoporotic patients than in non-osteoporotic individuals (Figure 3). Furthermore, the abundance of eight miRNAs, including miR-186-5p, miR-140-5p, miR-31-5p, miR-34-5p, miR-143-5p, miR-423-5p, miR-122-5p, and miR-542-5p, was significantly lower in osteoporotic patients than in non-osteoporotic individuals (Figure 3). These findings indicated that dysregulated vesicle non-coding small RNAs may be involved in osteoporosis development. In addition, the results of our study demonstrated that the mRNA levels of Wnt1, Wnt5a, Wnt7a, and Wnt9a were lower in osteoporosis-derived EVs than in non-osteoporosis-derived EVs (Figure 4), while DKK mRNA was up-regulated in osteoporotic individuals compared to non-osteoporotic individuals. Our results suggest that Wnt signalling is a potential regulator of osteoporosis development. Many studies demonstrated a role for the Wnt pathway in osteoporosis [30, 31]. When Wnt is active, adipogenic transcription factors are reduced expressed, and preadipocytes are thus maintained in an un-differentiated state [32]. Defective Wnt signalling in osteoporotic bone lead to impairment of tissue response, and further increased risk of fracture. β-catenin as the downstream effector of Wnt activation has a role in bone remodel and bone damage repair [33, 34], which further determined the bone mass distributions in the patients with osteoporosis.

Our study still has some limitations. First, small sample size and the results need independent validation with a larger cohort. In addition, which subtypes of EVs are responsible for the regulation of osteoporosis development remains to be investigated. Third, we have not assessed the *in vivo* function of EVs in osteoporosis with an animal model. We will extend the findings of this study to the effects of EVs on biochemical pathways known to regulate osteoclast maturation and hydroxyapatite metabolism (e.g., RANK, RANKL, Osteoprotegerin, PTH).

In conclusion, our pilot study demonstrated for the first time a significantly higher EV concentration in osteoporosis patients than in individuals without osteoporosis. There were significant alterations in EV cargo contents, including miRNAs and Wnt RNA molecules. More importantly, a functional assay indicated that osteoporotic-derived EVs notably decreased the mineralization capacity of osteoblasts. These findings provide potential novel strategies for therapeutic intervention.

## MATERIALS AND METHODS

### Patients and human sample collection

Written informed consent was received from all subjects. Plasma was obtained from 22 individuals without osteoporosis and 22 osteoporosis patients who were confirmed by standard dual energy X-ray absorptiometry examination. The recruited patients were diagnosed with osteoporosis based on radiographic or dual energy x-ray absorptiometry. BMD was calculated by a dedicated osteoporosis calibration phantom. All the patients were being treated with bisphosphonates during the study. This study was approved by the institutional review board at Zhejiang Provincial People’s Hospital.

### Animal model of osteoporosis

C57BL/6 female mice, ~16 weeks of age, were purchased from Shanghai Laboratory Animal Center. Fifteen mice (*n* = 15) were used to evaluate plasma EV concentration by using the animal model of unloading-induced osteoporosis. There are three groups: the age-matched control group (AC group, *n* = 5), hindlimb unloading group (HU group, *n* = 5), and salubrinal-treated hindlimb unloading group (US group, *n* = 5). To examine the pathogenesis of disuse osteoporosis, ten mice (HU and US) were subjected to hindlimb unloading for 2 weeks. C57BL/6J mice were subjected to hindlimb unloading by tail suspension. Subcutaneous administration was performed for salubrinal-treated group (salubrinal, 1 mg/kg body weight, Tocris Bioscience). The animal protocol (20170131A) was approved by Zhejiang Provincial People’s Hospital.

### ExoQuick™ EV isolation

EVs were isolated using ExoQuick Total Exosome Isolation Kit (System Bioscience Inc.) as described [35]. EVs were also recovered by using a differential ultracentrifugation protocol. EV pellets were solubilized with 1X PBS buffer and was utilized for further characterization.

### Transmission electron microscopy (TEM)

EVs were fixed in 2% paraformaldehyde solution and processed into ultrathin sections. All sections were examined by transmission electron microscopy. EV size distribution and concentration was evaluated using a NanoSight NS500.

### TaqMan miRNA assay

The candidate miRNAs for the experiment included miR-335-5p, -320a, -483-5p, -21-5p, -186-5p, -140-5p, -31-5p, -34-5p, -143-5p, -423-5p, -122-5p, and miR-542-5p. Total RNA was extracted from EVs by using an miRNeasy Serum/Plasma Kit (Qiagen). Real-time PCR was performed with TaqMan miRNA assay (Applied Biosystems). The relative fold change in each miRNA was calculated using the ΔΔCt method. U6 RNA was chosen as a consensus housekeeping miRNA for data normalization.

### Osteoblast mineralization

SaOS-2 cells (#ATCC HTB-85) were cultured in McCoy5A medium supplemented with 15% Fetal Bovine Serum (FBS, Gibco). To induce mineralization, the cells were induced with 50 μg/ml ascorbic acid and 10 mM β-glycerophosphate. At day 14, we employed osteogenesis assay kit (Millipore #ECM815) to quantify the mineralization.

### Western blotting

Briefly, 10 μg of total protein was denatured in 2%SDS loading buffer. Protein samples were subjected to separation on 12% Bis-Tris polyacrylamide gel, then transferred to polyvinylidene fluoride membrane, which was further probed with anti-Annexin V antibody (Abcam) and anti-TSG101 antibody (Abcam). Secondary antibody was Goat anti-rabbit conjugated with horseradish peroxidase (SantaCruz).

### Statistical analysis

Mann-Whitney test and Kruskal-Wallis test were chosen as indicated for data analysis. BMD correlation was analyzed by D’Agostino-Pearson test. GraphPad Prism 6.0 was used in this study. *P* < 0.05 was considered significant.

## Supplementary Materials

Supplementary Figure 1
