# Supplementary material for: Evaluation of the cargo contents and potential role of extracellular vesicles in osteoporosis
Source: Aging (Albany NY). 2021 Aug 10;13(15):19282–92. doi: 10.18632/aging.203264 (PMC8386556; doi:10.18632/aging.203264)
Supplement: Supplementary Figure 1 [file aging-13-203264-s001.pdf]

SUPPLEMENTARY FIGURE

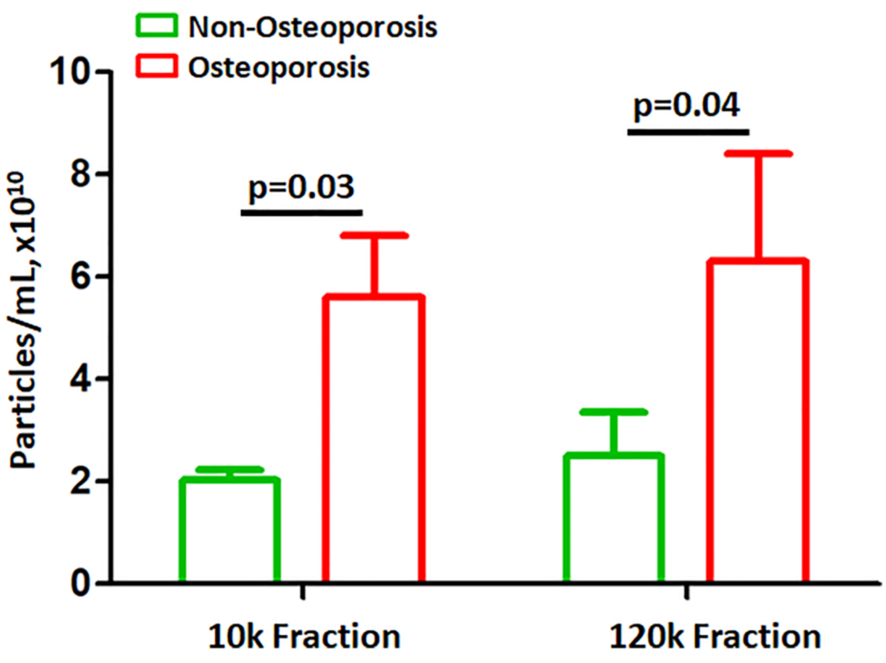

Supplementary Figure 1. NTA analysis of plasma EVs isolated from non-osteoporotic (*n* = 8) and osteoporotic (*n* = 8) individuals using differential ultracentrifugation. EVs were isolated from both the 10K and 120K fractions as indicated.
